# Osteoclastic expression of higher-level regulators NFATc1 and BCL6 in medication-related osteonecrosis of the jaw secondary to bisphosphonate therapy: a comparison with osteoradionecrosis and osteomyelitis

**DOI:** 10.1186/s12967-019-1819-1

**Published:** 2019-03-04

**Authors:** Falk Wehrhan, Christian Gross, Kay Creutzburg, Kerstin Amann, Jutta Ries, Marco Kesting, Carol-Immanuel Geppert, Manuel Weber

**Affiliations:** 1 Department of Oral and Maxillofacial Surgery, Friedrich-Alexander University Erlangen-Nürnberg, Glückstraße 11, 91054 Erlangen, Germany; 2 Department of Nephropathology, Institute of Pathology, Friedrich-Alexander University Erlangen-Nürnberg, Erlangen, Germany; 3 Institute of Pathology, Friedrich-Alexander University Erlangen-Nürnberg, Erlangen, Germany

**Keywords:** Medication related osteonecrosis of the jaw, MRONJ, BRONJ, Osteoradionecrosis, ORN, Osteomyelitis, Osteoclasts, NFATc1, BCL6, Osteoclastic regulators

## Abstract

**Background:**

With an increasing indication spectrum of antiresorptive drugs, the medication-related osteonecrosis of the jaw secondary to bisphosphonate therapy [MRONJ (BP)] is continuously gaining clinical relevance. Impaired osteoclast function, accompanied by altered cell morphology and expression of osteoclastic effector proteins, contributes to the pathogenesis of MRONJ (BP). However, the underlying regulatory mechanisms at a transcriptional level are unaddressed so far. These mechanisms are crucial to the development of disease-characteristic osteoclastic anomalies, that contribute to the pathogenesis of MRONJ (BP). NFATc1 is considered a master upstream osteoclastic activator, whereas BCL6 acts as osteoclastic suppressor. The present study aimed to elucidate the NFATc1 and BCL6 mediated osteoclastic regulation and activity in MRONJ (BP) compared to osteoradionecrosis (ORN) and osteomyelitis (OM) and normal jaw bone.

**Methods:**

Formalin-fixed jaw bone specimens from 70 patients [MRONJ (BP) n = 30; OM: n = 15, ORN: n = 15, control: n = 10] were analyzed retrospectively for osteoclast expression of NFATc1 and BCL6. The specimens were processed for H&E staining and immunohistochemistry. The histological sections were digitalized and analyzed by virtual microscopy.

**Results:**

Osteoclastic expression of NFATc1 and BCL6 was significantly higher in MRONJ (BP) specimens compared to OM and control specimens. NFATc1 and BCL6 labeling indices revealed no significant differences between MRONJ (BP) and ORN. The ratio of nuclear BCL6+ osteoclasts to cytoplasmic BCL6+ osteoclasts revealed significantly higher values for MRONJ (BP) specimens compared to OM and controls.

**Conclusion:**

This study displays that osteoclasts in MRONJ (BP) tissues feature increased expression of the higher-level regulators, paradoxically of both NFATc1 and BCL6. These observations can help to explain the genesis of morphologically altered and resorptive inactive osteoclasts in MRONJ (BP) tissues by outlining the transcriptional regulation of the pathomechanically relevant osteoclastic effector proteins. Furthermore, they strengthen the etiological delineation of MRONJ (BP) from OM and extend the osteoclast profiles of MRONJ (BP), OM and ORN and thus could lead to a better histopathological differentiation that can improve treatment decision and motivate new therapeutic concepts.

## Background

Osteonecrotic changes of the jaw bones can lead to severe functional and aesthetic limitations to the affected patient [1]. In addition to the osteoradionecrosis (ORN) and osteomyelitis (OM), the medication-related necrosis of the jaw secondary to bisphosphonate therapy [MRONJ (BP)] is increasing in incidence due to the extending indication spectrum of bisphosphonates (BP) [1]. Nowadays MRONJ (BP) represents the most common type of osteonecrosis of the jaw [2]. The clinical diagnosis of these three different types of destructive, inflammatory jaw bone disorders are made anamnestically, by clinical examination and radiologically. Although the clinical appearance of these diseases can be similar, they represent obligate distinguishable diseases with a different pathogenesis [1, 3, 4].

MRONJ (BP) [formerly: bisphosphonate-related osteonecrosis of the jaw (BRONJ)] is a serious side effect of the therapy with bisphosphonates that are indicated in conditions such as osteoporosis, multiple myeloma and osseous metastases of solid tumors. The umbrella term MRONJ, which also includes osteonecrosis caused by other antiresorptives (e.g. denosumab) and some antiangiogenic drugs, is currently defined by 3 mandatory parameters: 1. Exposed bone in the maxillofacial region that does not heal within 8 weeks after identification by a health care provider. 2. Exposure to an antiresorptive agent. 3. No history of radiation therapy to the craniofacial region [1].

ORN, on the other hand, represents a pathological bone condition which is predominantly associated to radiation doses > 60 Gy. The clinical manifestations of ORN, but also MRONJ (BP), are often due to secondary superinfection [5, 6].

The acute and the secondary chronic OM of the jaw are pathogen-induced infections of the bone marrow space, which can spread to the entire bone. The infection occurs endogenously (hematogenous scattering—usually monomicrobial) or exogenously (trauma or iatrogenic effects—mostly polymicrobial) [7].

Although the current treatment regimens of all three jaw-bone pathologies usually involve antimicrobial chemotherapy, surgical removal of necrotic bone and tight wound closure, they, however, differ in terms of adjuvant and alternative therapeutic options [1, 4, 7, 8].

The pathogenesis of MRONJ (BP), OM and ORN is poorly understood at the cellular level.

In particular, osteoclasts are at the center of research because they represent key cells of bone homeostasis [9]. Although comparative histopathological studies have already characterized bone morphology in these pathological conditions at the tissue level, studies analyzing osteoclastic parameters, such as quantity, have shown heterogeneous results, particularly with respect to osteoclasts in MRONJ (BP) tissues [10, 11]. Therefore, in our previous study, we systemically investigated and demonstrated differences of osteoclast profiles of MRONJ (BP), OM and ORN regarding osteoclast morphology, quantity, and the expression of the osteoclastic effectors dendritic cell-specific transmembrane protein (DC-STAMP) (associated to cell–cell fusion) and tartrate-resistant acid phosphatase (TRAP) (associated with osteoclastic bone resorption) [12].

In human MRONJ (BP) jaw bone samples we found giant, hypernucleated but resorptive inactive osteoclasts [12]. Disruption of the mevalonate metabolism, that is postulated to be the main effect of nitrogen-containing BPs on osteoclasts, does not provide an adequate explanation for the found osteoclastic anomalies [13]. Instead, the observed high expression of DC-STAMP and low expression of TRAP could help to explain them [12]. However, underlying regulatory mechanisms, especially those controlling the expression of the analyzed effector proteins and the cellular activity, remained unaddressed. Studies investigating the expression of the receptor activator of nuclear factor kappa-B ligand (RANKL) and the osteoclastic receptor activator of nuclear factor kappa-B (RANK) in MRONJ (BP) tissues have shown contradictory results [14–16]. Interestingly, it is known that the sole selective inhibition of RANKL by the monoclonal anti-RANKL antibody denosumab can cause MRONJ as well [17]. Although causing the same clinical manifestations, the pathomechanism of the denosumab-associated MRONJ and MRONJ (BP) differ as bisphosphonates, by disturbing the mevalonate metabolism, affect osteoclasts much more unspecifically and more complexly than denosumab [13, 17]. In order to delineate the osteoclastic regulation in MRONJ (BP) that takes place in-between RANK and the effector proteins already examined, the current study focused on the analysis of key transcription regulators, namely Transcription factor nuclear factor of activated T cell 1 (NFATc1) and B-cell lymphoma 6 protein (BCL6).

NFATc1, subordinated to RANK, is considered a master osteoclastic regulator, that is essential for osteoclastogenesis and osteoclastic activation [18]. NFATc1 plays a pivotal role in osteoclast fusion and osteoclast activation via up-regulation of various genes responsible for osteoclast adhesion, migration, acidification, degradation of inorganic and organic bone matrix, such as TRAP, DC-STAMP and Cathepsin K [19–21]. In osteoclasts RANK induces the NFATc1 gene via transcriptions factors such as nuclear factor-kappa B (NF-κB) and c-Fos [22]. RANK additionally activates phospholipase Cγ (PLCγ) and calcium signaling, which in turn can induce NFATc1 gene expression. Especially, the activation of calcium signaling leads to the recruitment of NFATc1 to its own promoter (autoamplification). NFATc1-deficient osteoclastic progenitor cells are unable to differentiate into osteoclasts, while ectopic expression of NFATc1 triggers osteoclastic differentiation also without RANKL [23]. This interface position between RANKL-dependent and RANKL-independent osteoclastic signaling pathways furthermore underlines the role of NFATc1 as a key osteoclastic regulator [18].

BCL6, a zinc finger transcriptional repressor, is usually associated with normal and malignant B cell development [24, 25]. In osteoclasts, BCL6 directly binds to the promoters of NFATc1, DC-STAMP and cathepsin K to suppress osteoclastogenesis and cellular activation. RANK, but also NFATc1, activate genes for transcriptional repressors, such as B lymphocyte-induced maturation protein-1 (Blimp1) in order to suppress BCL6 [26]. BCL6-deficient mice show an acceleration of osteoclast differentiation and develop severe osteoporosis [27].

The present study aimed to elucidate the status of cellular activity of osteoclasts in MRONJ (BP), OM, ORN and normal bone by analyzing formalin-fixed routine jaw bone specimens from patients regarding the osteoclastic expression of NFATc1 and BCL6. It was conducted to contribute to the understanding of osteoclastic regulation and activity alterations in MRONJ (BP) that might play a role in the pathogenesis. Furthermore, this study intended to contribute to the histopathological differentiation of MRONJ (BP), OM and ORN that can improve treatment decision and motivate new therapeutic concepts.

## Materials and methods

### Patients and tissue collection

The present study analyzed retrospectively jaw bone samples (n = 70) from 70 patients (MRONJ (BP): n = 30, ORN: n = 15, OM: n = 15, control: n = 10). Patient cohorts and respective samples from this study are identical to those in our previous study [12]. All patients were treated in the Department of Oral and Maxillofacial Surgery of the University Hospital Erlangen between 2007 and 2015. The analyzed jaw bone samples were gathered intraoperatively as part of routine histopathological diagnostics. The control group consisted of patients with histopathologically inconspicuous jaw bone samples (n = 10) which were obtained during dental surgery procedures of teeth with no signs of local infection. The jaw bone probes were fixed in 4% formalin immediately after surgical sampling. Histopathological analysis was performed by the Department of Pathology of the University Hospital Erlangen. Specific clinical disease criteria were checked by the review of medical records and radiographs.

Inclusion criteria for MRONJ (BP) samples were: (1) histopathological confirmation of MRONJ (BP). (2) Evidence of more than 8 weeks of exposed jaw bone. (3) Documented bisphosphonate therapy. (4) No radiotherapy. (5) No therapy with denosumab, bevacizumab, pazopanib, sunitinib, mTOR inhibitors and sorafenib.

Inclusion criteria for OM samples were: (1) histopathological confirmation of OM with evidence of chronic inflammatory processes in the jaw bone. (2) No bisphosphonate therapy. (3) No radiotherapy. Patients with primary chronic OM (non-bacterial cause) were excluded.

Inclusion criteria for ORN samples were: (1) Evidence of devitalized and exposed jaw bone in a previously irradiated field in the absence of local neoplastic processes. (2) No bisphosphonate therapy.

Inclusion criteria for control samples were: (1) no histopathologic evidence of bone disease. (2) No bisphosphonate or local radiation therapy. (3) No medications significantly affecting jaw bone homeostasis. (4) No intraoral inflammation. (5) No relevant periodontitis. (6) No local malignancies. (7) No relevant systemic diseases (e.g., osteoporosis).

For detailed patient data, see Table 1.

**Table 1:** Patient data

	Number of patients	Sex	Age (years)	(Primary) diagnosis	Extraction location	Additional information	Smoking status
MRONJ (BP)	30	53.3% women (16)	Ø 67.8 ± 8.89	33.33% prostate cancer (10), 30% breast cancer (9), 20% multiple myeloma (6),10% osteoporosis (3), 0.33% renal cell carcinoma (1), 0.33% vertebral sclerosis (1)	76.7% lower jaw (23), 23.3% upper jaw (7)	100% nitrogenous. BPs (30): 70% zoledronate (21), 13.3% alendronate (4), 6.6% risedronate (2), 6.6% ibandronate (2), 3.3% pamidronate (1)	13 smoker, 13 non-smoker, 4 unknown
OM	15	53.3% women (8)	Ø 43.6 ± 25.20	86.6% chronic osteomyelitis (13), 13.3% acute osteomyelitis (2)	100% lower jaw (15)		6 smoker, 6 non-smoker, 3 unknown
ORN	15	13.3% women (2)	Ø 57 ± 7.89	60% SCC oral cavity (9), 13.3% SCC oropharynx (2), 6.6% SCC hypopharynx (1), 6.6% SCC tonsil (1), 6.6% SCC cranial skin (1), 6.6% CUP	100% lower jaw (15)	Ø total reference dose in the mandibular region: 68 Gy. (The applicated dose was set individually by the radiotherapists)	11 smoker, 2 non-smoker, 2 unknown
CONTROL	10	40% women (4)	Ø 33.8 ± 16.17	50% facial fracture (5), 20% dysgnathia (2), 10% cleft lip and palate (1), 10% wisdom tooth extraction (1), 10% arch ratio anomaly (1)	80% lower jaw (8), 20% upper jaw (2)		2 smoker, 3 non-smoker, 5 unknown

*Ø* mean, *min* minimum, *max* maximum, *BP* bisphosphonate, *MRONJ (BP)* medication-related osteonecrosis of the jaw secondary to bisphosphonate therapy, *OM* osteomyelitis, *ORN* osteoradionecrosis, *SCC* squamous cell carcinoma, *CUP* cancer of unknown primary. The examined patient cohorts were also used in our previous study [12]

### Immunohistochemical staining

All formalin-fixed samples underwent decalcification and were embedded in paraffin before being sliced in 3-μm sections using a microtome (RM2165, Leica, Nussloch, Germany). Special microscope slides with improved adhesion were used (SUPERFROST ULTRA PLUS, Gerhard Menzel GmbH, Braunschweig, Germany). The sections underwent dewaxing in xylene and rehydration in graded propanol and distilled water before staining.

Hematoxylin and eosin staining (H&E) was carried out according to standard protocols.

Immunohistochemistry was performed using an automated staining device (Autostainer plus, DakoCytomation, Dako Deutschland GmbH, Hamburg, Germany). Antigen retrieval consisted of section treatment with ethylenediaminetetraacetic acid (EDTA) (dilution 1:100, PMB4-125, Antigen Retrieval Buffer 4, Spring Bioscience, CA, USA) at 66.7 °C for 5 h. The reduction of background staining artifacts was achieved by performing peroxidase-blocking for 5 min (S2023, DAKO REAL, Peroxidase-Blocking Solution, Dako Deutschland GmbH, Hamburg, Germany).

The following primary antibodies were used for protein detection:

- Anti-NFATc1-AK (sc-7294, NFATc1 (7A6), mouse, monoclonal, Santa Cruz Biotechnology, Inc., Heidelberg, Germany). Dilution: 1:50. Incubation time: 20 min.
- Anti-BCL6-AK (HPA004899, Anti-BCL6, rabbit, polyclonal, Atlas Antibodies AB, Stockholm, Sweden). Dilution: 1:50. Incubation time: 20 min.

EnVision Detection System Peroxidase/diaminobenzidine (DAB), Rabbit/Mouse (K5007 HRP/DAB+, Dako Deutschland GmbH, Hamburg, Germany) was used as staining kit. This kit provided a conjugated dextran and a DAB+ chromogen that were used for visualizing the antibody-marked proteins. Hematoxylin was used for nuclear counterstaining (CS700, Dako Deutschland GmbH, Hamburg, Germany). Positive and negative controls were included in each staining series.

### Quantitative immunohistochemical analysis

**Fig. 1:**
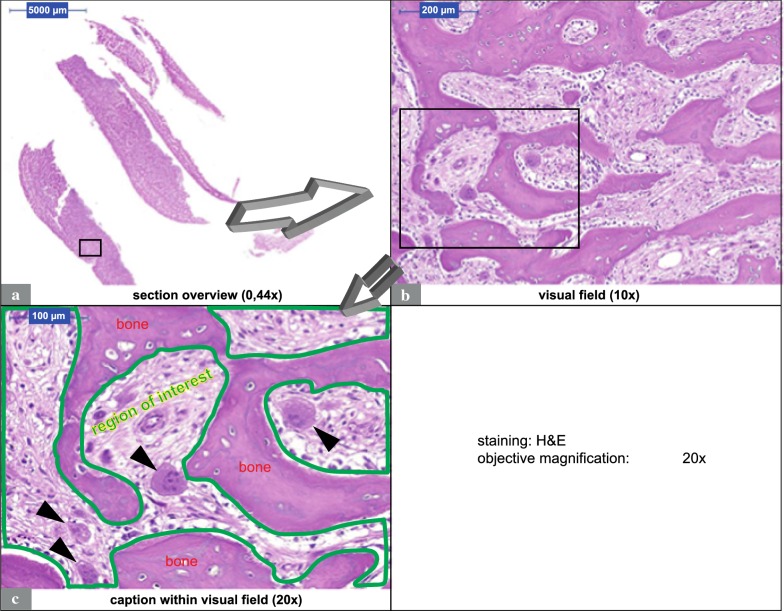
Whole slide Imaging. **a**–**c** illustrate the procedure of virtual microscopy. The figure shows an H&E stained MRONJ (BP) section. Section scanning was performed by using a Pannoramic 250 Flash III Scanner (3DHISTECH Kft., Budapest, Hungary). Pannoramic Viewer (3DHISTECH Kft., Budapest, Hungary) was used for virtual microscopy. Visual fields were set within the scanned sections (**a** rectangle). **b** shows a visual field. **c** Caption within a visual field. Regions of interest were marked and the included area was determined (**c** area within green line). Arrows tag the location of osteoclasts

All stained histological sections were scanned and digitalized completely in cooperation with the Institute of Pathology of the University Hospital Erlangen using a Pannoramic 250 Flash III Scanner (3DHISTECH Kft., Budapest, Hungary). Before scanning, the sections were quality-checked under a bright-field microscope (Axioskop, Zeiss, Jena, Germany; at a magnification of 100×–400×). The analysis of the digitalized sections was done via virtual microscopy using CaseViewer version 2.2 (3DHISTECH Kft., Budapest, Hungary). Figure 1 illustrates the method of “whole slide imaging”, that was used in this study. Two visual fields per virtualized section were set within areas with a high probability for the presence of osteoclasts (bone trabeculae, subperiosteal bone, endosteal structures and connective tissue directly adjacent to the bone). If the visual field size exceeded the total section size, only one visual field was used. Areas of pure necrosis were omitted for analysis. Within the visual fields, non-bony medullary areas were marked (regions of interest = ROIs) (Fig. 1c). Any cell counting occurred only within ROIs. Cells were considered osteoclasts if they met the following morphological criteria: (1). Multinuclearity (at least two nuclei). (2). Large cell body (larger than two fused mononuclear cells). (3). Direct contact with bone or proximity to bone. (4). No proximity to granulomatous foci or foreign particles. Area determination within the visual fields was done with Pannoramic Viewer, whereas cell counting was performed with ImageJ (Rasband, W.S., ImageJ, US National Institutes of Health, Bethesda, Maryland, USA, http://imagej.nih.gov/ij/, 1997–2014). Section analysis was conducted by two medical students familiar with tissue morphology, IHC-methods and analysis. These students were blinded to the origin of the specimens. Regarding cell counting, inter-individual differences were checked and did not exceed 10%.

### Statistical analysis

Statistical analysis was conducted after consultation with the Department of Medical Informatics, Biometry and Epidemiology (IMBE) of the Friedrich-Alexander University Erlangen-Nürnberg.

For quantitative analysis, not only the ratios of osteoclasts to ROI were determined, but also the respective labeling indices (positive osteoclasts of a ROI/all osteoclasts of a ROI) and the ratio of nuclear BCL6+ osteoclasts to cytoplasmic BCL6+ osteoclasts. Results are expressed as the minimum, maximum, average, median, interquartile range (IQR) and standard deviation (SD). Box plot diagrams visualize the respective values.

The Kolmogorov–Smirnov test was used for normal distribution testing. The Mann–Whitney U test was used for statistical hypothesis testing. P-values ≤ 0.05 were considered statistically significant. SPSS 22 (SPSS, IBM, New York, USA) was used for statistics.

## Results

### Osteoclastic NFATc1 expression patterns

**Fig. 2:**
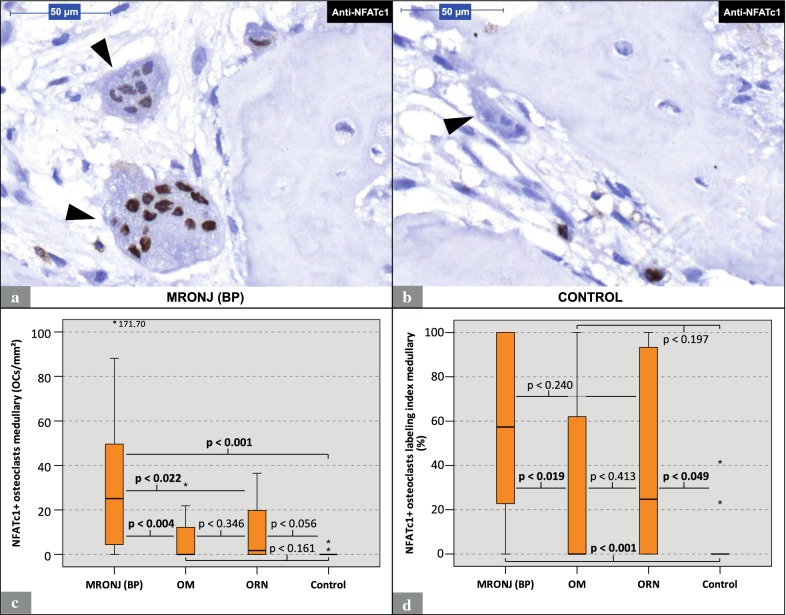
Analysis of anti-NFATc1 staining: MRONJ (BP) vs. OM vs. ORN vs. control. **a** Visual field from a MRONJ (BP)-specimen with two giant multinucleated OCs with stained NFATc1+ nuclei (arrowheads). Note that this staining predominantly stains nuclei. **b** Visual field from a control specimen with an unstained OC (arrowhead). The specimens in **a** and **b** underwent anti-NFATc1 staining under the same conditions. **c** Visualizes the number of NFATc1+ OCs per ROI for each group. **d** Illustrates the respective labeling indices. *Marks statistical outliers. For detailed data see Table 2. *MRONJ (BP)* medication-related osteonecrosis of the jaw secondary to bisphosphonate therapy, *OC* osteoclasts, *OM* osteomyelitis, *ORN* osteoradionecrosis, *NFATc1* nuclear factor of activated T-cells, cytoplasmic 1; *ROI* region of interest

NFATc1-positive (NFATc1+) cells showed a brown nuclear staining (Fig. 2a). NFATc1-expression occurred not only in osteoclasts, but also in mononuclear cells (Fig. 2a, b). The expression of NFATc1 in osteoclasts was restricted to the cell nuclei. NFATc1+ osteoclasts were found in specimens from all groups. The amount of NFATc1+ osteoclasts per ROI were found to be significantly higher in MRONJ (BP) specimens (median: 25.1 osteoclasts/mm^2^) than in ORN (median: 1.8 osteoclasts/mm^2^; p < 0.022), OM (median: 0.0 osteoclasts/mm^2^; p < 0.004), and control specimens (median: 0.0 osteoclasts/mm^2^; p < 0.001) (Table 2; Fig. 2c). The analysis of the osteoclast NFATc1 labeling indices revealed that MRONJ (BP) specimens (median: 57.3%) featured significantly higher indices than OM (median: 0.0%; p < 0.019) and control specimens (median: 0.0%; p < 0.001) (Table 2; Fig. 2d). However, no significant differences between the osteoclast NFATc1 labeling indices of MRONJ (BP) (median: 57.3%) and ORN specimens (median: 24.8%; p < 0.240) were found. For detailed data, see Table 2.

**Table 2:** Descriptive data

	Group	Min	Max	Mean	Median	IQR	SD
Anti-NFATc1 staining							
Nuclear expression							
NFATc1 + osteoclasts per ROI (osteoclasts/mm^2^)	MRONJ (BP)	0.0	171.7	31.2	25.1	45.9	36.1
	OM	0.0	31.4	7.0	0.0	14.1	9.8
	ORN	0.0	36.5	11.4	1.8	22.3	13.8
	CONTROL	0.0	5.8	0.8	0.0	0.6	1.9
Labeling index (%/100)	MRONJ (BP)	0	1	0.569	0.573	0.790	0.381
	OM	0	1	0.304	0	0.650	0.396
	ORN	0	1	0.424	0.248	1	0.440
	CONTROL	0	0.420	0.065	0	0.060	0.144
Anti-BCL6 staining							
Cytoplasmic expression							
BCL6 + osteoclasts per ROI (osteoclasts/mm^2^)	MRONJ (BP)	0.0	113.1	33.2	29.1	29.4	25.9
	OM	0.0	43.4	11.4	9.2	16.5	12.4
	ORN	0.0	141.3	19.7	5.6	13.6	35.8
	CONTROL	0.0	9.2	1.7	0.0	3.5	3.1
Labeling index (%/100)	MRONJ (BP)	0	1	0.760	0.860	0.380	0.300
	OM	0	1	0.445	0.444	0.860	0.391
	ORN	0	1	0.714	0.839	0.560	0.349
	CONTROL	0	0.370	0.100	0	0.300	0.163
Nuclear expression							
BCL6 + osteoclasts per ROI (osteoclasts/mm^2^)	MRONJ (BP)	0	73.4	17.6	13.1	21.4	16.9
	OM	0	11.4	2.9	2.1	4.1	3.7
	ORN	0	92.5	10.8	2.5	10.4	23.4
	CONTROL	0	9.3	1.9	0	4.0	3.3
Labeling index (%/100)	MRONJ (BP)	0	1	0.469	0.483	0.58	0.329
	OM	0	0.72	0.160	0.075	0.29	0.218
	ORN	0	1	0.416	0.282	0.46	0.342
	CONTROL	0	0.56	0.109	0	0.27	0.189
Nuclear BCL6 + osteoclasts to cytoplasmic BCL6 + osteoclasts (%/100)	MRONJ (BP)	0	1	0.513	0.503	0.433	0.308
	OM	0	0.450	0.164	0.127	0.355	0.177
	ORN	0	0.778	0.460	0.500	0.333	0.237
	CONTROL	0	1	0.243	0	0.550	0.424

*Min* minimum, *Max* maximum, *IQR* interquartile range, *SD* standard deviation, *ROI* region of interest, *MRONJ (BP)* medication-related osteonecrosis of the jaw secondary to bisphosphonate therapy, *OM* osteomyelitis, *ORN* osteoradionecrosis, *NFATc1* nuclear factor of activated T-cells, cytoplasmic 1, *BCL6* B-cell lymphoma 6

### Osteoclastic BCL6 expression patterns

**Fig. 3:**
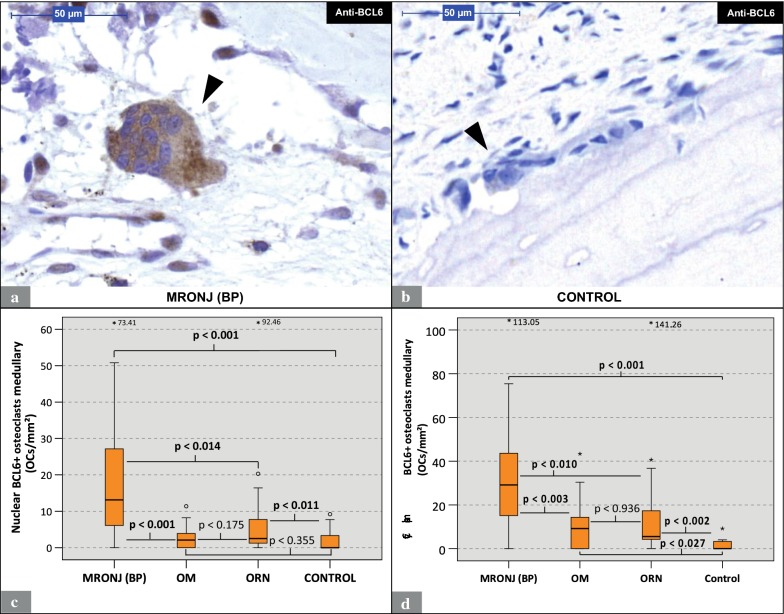
Analysis of anti-BCL6 staining (Part 1): MRONJ (BP) vs. OM vs. ORN vs. control. **a** Visual field from a MRONJ (BP)-specimen. Note the giant multinucleated OC (arrowhead) with cytoplasmic expression of BCL6 and the surrounding mononuclear cells with nuclear BCL6-expression. **b** Visual field from a control specimen with an unstained OC (arrowhead). The specimens in **a** and **b** underwent anti-BCL6 staining under the same conditions. **c** Visualizes the number of OCs with nuclear BCL6+ expression per ROI for each group. **d** Illustrates the number of OCs with cytoplasmic BCL6+ expression per ROI for each group. *Marks statistical outliers. For detailed data see Table 2. *MRONJ (BP)* medication-related osteonecrosis of the jaw secondary to bisphosphonate therapy, *OC* osteoclasts, *OM* osteomyelitis, *ORN* osteoradionecrosis, *BCL6*, B-cell lymphoma 6, *ROI* region of interest

BCL6-positive (BCL6+) cells showed a brown nuclear and cytoplasmic staining (Fig. 3a). BCL6-expression occurred not only in osteoclasts, but also in mononuclear cells (Fig. 3a). BCL6+ osteoclasts were found in specimens from all groups. The quantitative analysis of the nuclear expression revealed a significantly higher ratio of BCL6+ osteoclasts per ROI in MRONJ (BP) specimens (median: 13.1 osteoclasts/mm^2^) than in ORN (median: 2.5 osteoclasts/mm^2^; p < 0.014), OM (median: 2.1 osteoclasts/mm^2^; p < 0.001) and control specimens (median: 0.0 osteoclasts/mm^2^; p < 0.001) (Table 2; Fig. 3c). The quantitative analysis of the cytoplasmic expression revealed the same patterns as for nuclear expression with a significantly higher ratio of BCL6 + osteoclasts per ROI in MRONJ (BP) specimens (median: 29.1 osteoclasts/mm^2^) than in ORN (median: 5.6 osteoclasts/mm^2^; p < 0.010), OM (median: 9.2 osteoclasts/mm^2^; p < 0.003) and control specimens (median: 0.0 osteoclasts/mm^2^; p < 0.001) (Tbl. 2; Fig. 3d).

**Fig. 4:**
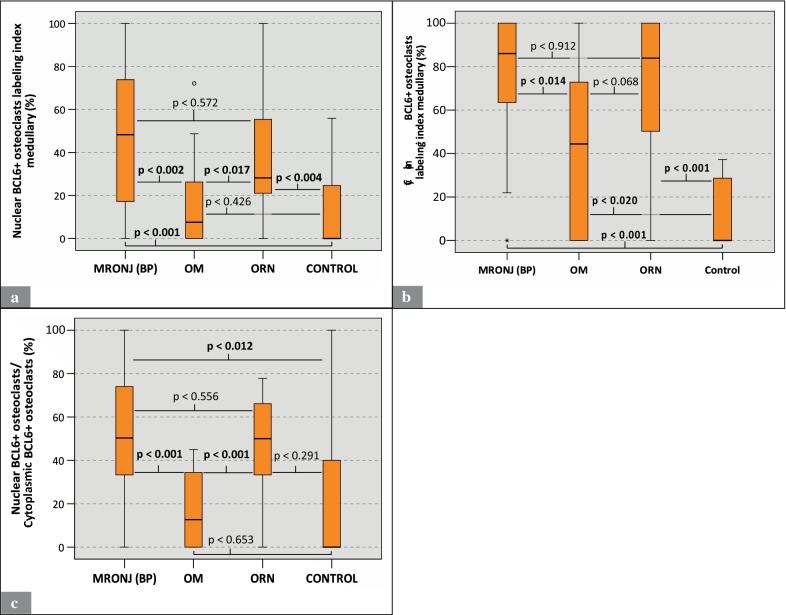
Analysis of anti-BCL6 staining (Part 2): MRONJ (BP) vs. OM vs. ORN vs. control. **a** Labeling index of OCs with nuclear expression of BCL6 (Nuclear BCL6 + OCs/all OCs). **b** Labeling index of OCs with cytoplasmic expression of BCL6 (Cytoplasmic BCL6 + OCs/all OCs). **c** Illustrates the ratio of nuclear BCL + OCs to cytoplasmic BCL + OCs. For detailed data see Table 2. *MRONJ (BP)* medication-related osteonecrosis of the jaw secondary to bisphosphonate therapy, *OC* osteoclasts, *OM* osteomyelitis, *ORN* osteoradionecrosis, *BCL6* B-cell lymphoma 6, *ROI* region of interest

The analysis of the osteoclast BCL6 labeling indices both for nuclear and cytoplasmic expression revealed that MRONJ (BP) specimens (nuclear median: 48.3%; cytoplasmic median: 86%) featured significantly higher indices than OM (nuclear median: 7.5%; cytoplasmic median: 44.4%; nuclear: p < 0.002; cytoplasmic: p < 0.014) and control specimens (nuclear median: 0.0%; cytoplasmic median: 0.0%; nuclear: p < 0.001; cytoplasmic: p < 0.001) (Table 2; Fig. 4a, b). However, no significant differences between the osteoclast BCL6 labeling indices of MRONJ (BP) and ORN (nuclear median: 28.2%; cytoplasmic median: 83.9%; nuclear: p < 0.572; cytoplasmic: p < 0.912) specimens were found (Table 2; Fig. 4a, b).

The calculation of the ratio of nuclear BCL6+ osteoclasts to cytoplasmic BCL6+ osteoclasts revealed significantly higher values for MRONJ (BP) specimens (median: 50.3%) than for OM (median: 12.7%; p < 0.001) and control specimens (median: 0.0%; p < 0.012) (Table 2; Fig. 4c). For detailed data, see Table 2. For p-values, see Figs. 3 and 4.

## Discussion

To our knowledge, this study is the first to immunohistochemically investigate the osteoclastic expression of NFATc1 and BCL6 in human jaw bone samples from patients with MRONJ (BP), ORN, and OM. The results of this study indicate that in MRONJ (BP) tissues the osteoclastic expression of NFATc1 and BCL6 is significantly increased compared to OM and control tissues (Table 2; Figs. 2, 3, 4). NFATc1 is considered a master regulator and activator of a variety of genes that are essential for osteoclastogenesis (e.g. DC-STAMP) and function (e.g. TRAP). Currently it is assumed that NFATc1 enhances the expression of genes coding for DC-STAMP and TRAP [20, 23, 28]. BCL6, contrary, is considered a suppressor of NFATc1-dependent genes, including DC-STAMP and TRAP [27]. For interpretation of the expression patterns of the higher-level regulators NFATc1 and BCL6, the findings of our previous study, which examined the expression of the osteoclastic effectors (DC-STAMP & TRAP) and the osteoclastic morphology of the same specimens from the same patients, can be helpful [12]. For this purpose, Table 3 summarizes the results of all examined parameters including those from the previous study. These data indicate that elevated osteoclastic expression rates of NFATc1 are accompanied by elevated osteoclastic expression rates of DC-STAMP (Fig. 2; [12]). The indicated co-expression, as seen in MRONJ (BP) and ORN tissues, may be explained by the postulated role of NFATc1 as an activator of DC-STAMP and osteoclastic regulation [23]. However, throughout all study groups no positive correlation was found between osteoclastic NFATc1 and TRAP expression (Fig. 2; [12]). Consistent with the currently postulated inhibitory role of BCL6 in RANK-dependent signaling [27], we observed an inverse relationship between BCL6 and TRAP expression. Throughout all study groups, elevated osteoclastic expression rates of BCL6 were accompanied by low osteoclastic expression rates of TRAP (Figs. 3, 4; [12]). However, osteoclastic BCL6 expression did not appear to be associated with a general inhibition of DC-STAMP expression as expected. Even a positive correlation between osteoclastic BCL6 and DC-STAMP expression was evident (Figs. 3, 4; [12]).

**Table 3:**
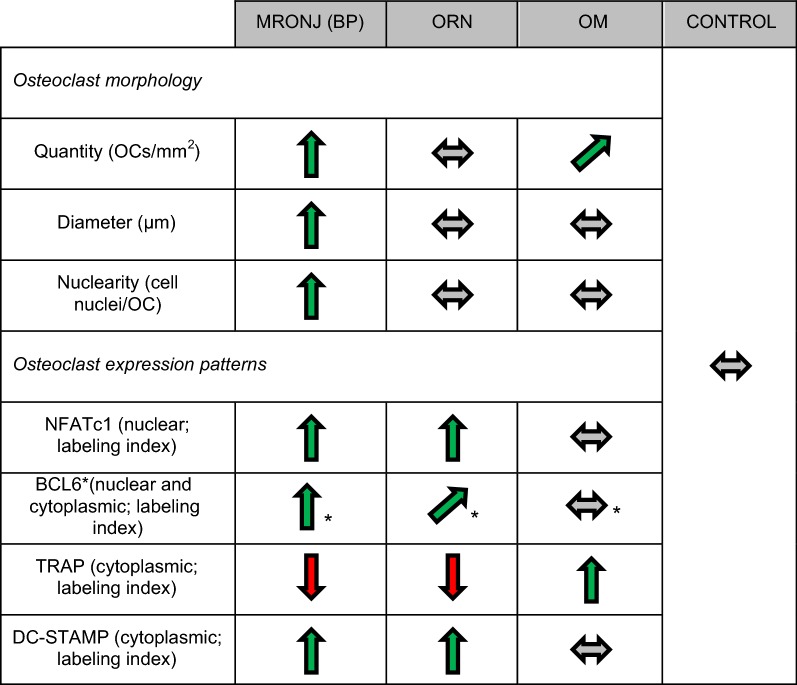
Overview: Osteoclast profiles

This chart summarizes the current and the past results of our work, that investigated the osteoclast profiles of MRONJ (BP), ORN, OM and CONTROL specimens. The arrows show the deviation from the values of the control group

*BP* bisphosphonate, *MRONJ (BP)* medication-related osteonecrosis of the jaw secondary to bisphosphonate therapy, *OM* osteomyelitis, *ORN* osteoradionecrosis, *TRAP* tartrate-resistant acid phosphatase, *BCL6* B-cell lymphoma 6 protein, *NFATc1* Transcription factor nuclear factor of activated T cell 1, *DC-STAMP* dendritic cell-specific transmembrane protein, *OC* osteoclast

* Indicates that the results for nuclear and cytoplasmic expression are pooled

The results of the present and previous study indicate expression combinations of the higher-level regulators and their downstream effectors that are partially not in line with current assumptions on osteoclastic regulatory mechanisms [20, 23, 27, 28]. On the one hand, it appears congruent with these assumptions that an increase in NFATc1 expression triggers DC-STAMP expression and an increased expression of BCL6 is associated with an inhibition of TRAP expression. However, on the other hand, it remains unclear why an increased expression of NFATc1 does not upregulate TRAP expression and an increased expression of BCL6 does not diminish DC-STAMP expression (Table 3). Furthermore, it appears paradox that MRONJ (BP) and ORN tissues feature an increased expression of both osteoclastic activator (NFATc1) and suppressor (BCL6). However, it needs to be considered that this study examined osteoclasts in pathological conditions. The disturbance of the mevalonate metabolism by bisphosphonates (MRONJ (BP)) [29], damage to the DNA by radiation (ORN) and external cellular stimulation by microbial factors (OM) usually lead to alterations of osteoclastic regulation and signaling [30, 31]. Thus, noxae-associated affections of osteoclasts might lead to partially altered osteoclastic regulatory mechanisms.

### Why does BCL6 also appear in the cytoplasm?

Although BCL6, a member of the POZ/BTB-zinc finger protein family, fulfills its function as a transcriptional repressor at a nuclear site [27], the immunohistochemical analysis of the present study revealed the presence of BCL6 in the cell nucleus as well as in the osteoclast cytoplasm (s. Figure 3a). Therefore, the nuclear and cytoplasmic expression of BCL6 expression in osteoclasts was studied separately and the ratio of nuclear to cytoplasmic expression was calculated. The results of nuclear and cytoplasmic BCL6 expression revealed no appreciable differences (Figs. 3, 4). In contrast, NFATc1 was found predominantly in the cell nuclei (Fig. 2a). The occurrence of BCL6 in both cell compartments was also observed in microadenoma and colorectal cancer cells [32]. In general, protein translation takes place predominantly in the extranuclear cell compartment [33]. However, for active suppression of genes, BCL6 must be translocated back into the cell nucleus. Thus, the presence of BCL6 in both cell compartments might be due to a prolonged retention time in the osteoclast’s cytoplasm. The retention time of BCL6 in the osteoclast’s cytoplasm seems to be much longer than that of NFATc1, since NFATc1 could only be detected in nuclei (Fig. 2a).

### Relevance of the findings for the understanding of MRONJ (BP) and its differential diseases

The analysis of the osteoclastic expression of NFATc1 and BCL6 allows deeper insights into the status of osteoclasts in the MRONJ (BP)-, ORN- and OM-affected jawbone.

We showed that the osteoclast profile of MRONJ (BP) is characterized by an increased osteoclastic expression of higher-level regulators, paradoxically both enhancer (NFATc1) and suppressor (BCL6) (Figs. 3, 4). In addition, the predecessor study elucidated that the osteoclast profile of MRONJ (BP) is further dominated by a high cell quantity, special cell morphology (giant hypernucleated osteoclasts), resorptive inactivity (TRAP expression low) and an increased cell–cell fusion rate (DC-STAMP expression high) [12]. In literature, the assumption that bisphosphonates inactivate and decimate osteoclasts is widespread [34–36]. This may be true for the bisphosphonate-exposed bone, but need to be put into perspective for MRONJ (BP), as osteoclasts in MRONJ (BP)-tissues can only be considered compromised in terms of their resorptive capacity (TRAP expression low) but not related to their overall cellular activity [12]. A general cellular inactivation of these osteoclasts cannot be assumed as “driving” regulatory mechanisms seem to be active (e.g. high expression of NFATc1).

The comparison between MRONJ (BP) and ORN osteoclast profiles revealed that these bone conditions are similar in terms of the expression patterns of NFATc1 and BCL6 (Figs. 2, 3, 4). However, as we could show previously, these entities differ significantly in terms of osteoclast quantity and morphology [12]. Osteoclasts in MRONJ (BP) tissue, although resorptive inactivate, seem to react to “driving” intracellular signals, such as an increased expression of NFATc1, by forming giant and hypernucleated cells. Contrary, radiation-affected osteoclasts in ORN tissues do not morphologically respond to an increased NFATc1 expression. This indicates that these pathologies, although clinically very similar, differ at the level of osteoclastic regulation.

After being first described in 2003, MRONJ (BP) was temporary not clearly distinguished from a type of OM [37]. However, a clear distinction between these two pathologies is mandatory as we could demonstrate that the osteoclast profiles of MRONJ (BP) and OM differ (Figs. 2, 3, 4, [12]). The increased expression of NFATc1 and the consecutive activation of DC-STAMP expression in MRONJ (BP) osteoclasts could occur as part of a compensatory reaction to the bisphosphonate-induced inhibition of the osteoclasts resorptive capability (TRAP expression low). In contrast, osteoclasts in OM tissue remain resorptive active (TRAP expression high) and thus may not require compensatory upregulation of NFATc1. This could explain the low osteoclast expression of NFATc1 in OM (Figs. 2, 3, 4). However, the observed differences in osteoclastic NFATc1 and BCL6 expression between MRONJ (BP) and OM tissues further strengthens the etiological delineation of these osteopathologies.

### From bench-to-bedside?

Clinically but also histopathologically, the differentiation of MRONJ (BP), OM and ORN can be difficult. It requires reliable medical history and clinical information to make the correct diagnosis. The study of the osteoclastic expression of the higher-level regulators NFATc1 and BCL6 not only deepens the understanding of the activity of osteoclasts in these bone conditions, but also extends the profiling possibilities of these key cells. By examining and compiling the osteoclast profiles of the relevant diseases, histopathological examination could gain significance and importance in distinguishing these diseases and thus contribute better to a correct therapeutic decision. A better understanding of the pathophysiologic cellular signaling in jaw bone osteonecrosis is a prerequisite to identify targeted therapeutic approaches.

### Limitations

The examined samples are from intraoperatively obtained routine biopsies. It follows that this study did not investigate standardized collectives and samples. Patient-dependent lifestyle factors affecting bone homeostasis, e.g. diet, exercise, and the age were not matched. The localization of the sampling within the jawbone also varied individually.

## Conclusion

The present study shows that osteoclasts in MRONJ (BP) tissues feature increased expression of the higher-level regulators, paradoxically both of the enhancer NFATc1 and the repressor BCL6. These observations can help to explain the genesis of morphologically altered and resorptive inactive osteoclasts in MRONJ (BP) tissues by depicting the transcriptional regulation of the pathomechanically relevant osteoclastic effector proteins. A general cellular inactivation of osteoclasts in MRONJ (BP)-tissues, despite the proven resorptive inactivity, cannot be assumed as these “driving” regulatory mechanisms seem to be active. The observation that osteoclasts in OM-tissues did not feature increased expression of NFATc1 and BCL6 further strengthens the etiological delineation of MRONJ (BP) from OM. Furthermore, the results of this study extend the osteoclast profiles of MRONJ (BP), ORN and OM and thus could contribute to a better histopathological differentiation and to a right treatment decision and potentially contribute to the identification of targeted therapies.

## Abbreviations

BCL6: B cell lymphoma 6 protein
DC-STAMP: dendritic cell-specific transmembrane protein
H&E: hematoxylin & eosin staining
MRONJ (BP): medication-related osteonecrosis of the jaw secondary to bisphosphonate therapy
NFATc1: transcription factor nuclear factor of activated T cell 1
OM: osteomyelitis
ONJ: osteonecrosis of the jaw
ORN: osteoradionecrosis
RANK: receptor activator of nuclear factor kappa-B
RANKL: receptor activator of nuclear factor kappa-B ligand
ROI: region of interest
TRAP: tartrate-resistant acid phosphatase
